# Consolidation and Permeability of the B1 and D1 Gas Hydrate Bearing Sands and Associated Seal Sediments of the Extended-Duration Gas Production Test Site on the Alaska North Slope

**DOI:** 10.1021/acs.energyfuels.5c03159

**Published:** 2026-05-07

**Authors:** Imgenur Tepecik, Yongkoo Seol, William F. Waite, Sheng C. Dai

**Affiliations:** † 1372 Georgia Institute of Technology, Atlanta, Georgia 30332, United States; ‡ 97012 National Energy Technology Laboratory, Morgantown, West Virginia 26507, United States; § United States Geological Survey, Woods Hole, Massachusetts 02543, United States

## Abstract

Gas hydrate, a solid combination of gas (mostly methane in nature) and water molecules stable at low temperatures and elevated pressures, occurs naturally in marine and permafrost-associated environments. Gas hydrate reservoirs, such as those in the Alaska North Slope, have been considered potential energy resources for gas production. To understand the petrophysical and geo-mechanical characteristics of the reservoir, core samples retrieved from the site of the JOGMEC-DOE-USGS collaborative gas hydrate R&D project have been analyzed in the laboratory for their hydraulic and mechanical properties. This paper focuses on both seal and reservoir samples associated with the B1 and D1 sands, which are evaluated for index properties (including porosity, grain size distribution, liquid and plastic limits, specific surface area, and specific gravity), consolidation, permeability, and water retention. Furthermore, the reservoir core samples were tested with pore-filling, laboratory-grown tetrahydrofuran hydrate, in order to assess reservoir behavior during gas production from hydrates. Under simulated in situ stress conditions, the seal and hydrate-free reservoir cores had a permeability anisotropy ratio of *k*_h_/*k*_v_ = 3.0–5.0, and *k*_h_/*k*_v_ = 2.4–3.0 for the reservoir tetrahydrofuran hydrate-bearing cores. The data suggest that depressurizing the reservoir to induce hydrate dissociation alters the reservoir effective permeability in three ways: permeabilities decrease due to porosity lost (e.g., the initial reservoir thickness can decrease by up to 5% upon 7 MPa depressurization), permeability increases due to the loss of solid hydrate in the pore space, and permeability anisotropy *k*_h_/*k*_v_ decreases in response to the evolving pore-space geometry. We show that given the simulated in situ gas hydrate saturations (i.e., *S*_h_ = 32% in core 7P-2E and *S*_h_ = 21% in core 20P-4), gas production from the dissociation of tetrahydrofuran hydrate in the two tested cores results in a net increase in effective permeability and a decrease in *k*_h_/*k*_v_. This study highlights the importance of investigating seal and reservoir sediments and the impacts of depressurization on the porosity and permeability responses during production.

## Graphical abstract

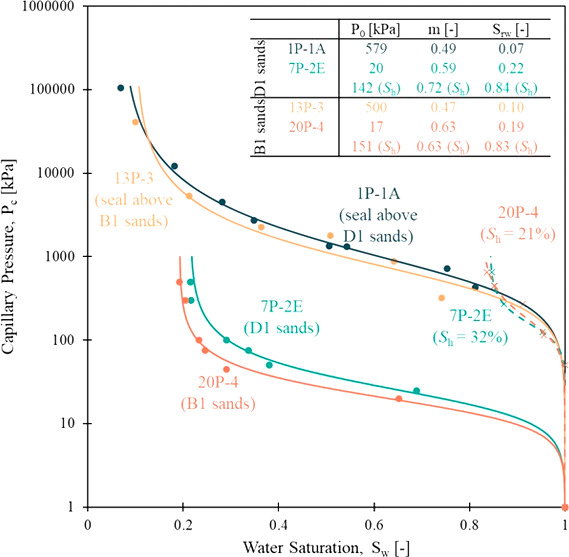

## 1 Introduction

For decades, gas hydrate reservoirs have been considered potential energy sources of the future.^1^ However, the reservoir response during gas production remains one of the biggest uncertainties in utilizing these resources.^2,3^ To directly investigate a reservoir’s response to depressurization and the production of gas and associated water, the U.S. Department of Energy (DOE) and the MH21-S R&D Consortium of Japan collaborated to run an extended-duration gas production test in the western Prudhoe Bay Unit, Alaska North Slope (ANS).^4^ Based on data collected from the project’s first phase, the HYDRATE-01 Stratigraphic Test Well (STW) was drilled in December 2018, and promising hydrate-bearing reservoir sediments were identified.^5^ The second phase of the project, the JOGMEC, DOE, and the U.S. Geological Survey (USGS) Collaborative Gas Hydrate R&D Project in Alaska, established the HYDRATE-02 Geo Data Well (GDW) in October 2022, which included the collection of pressure cores from the reservoir and bounding non-reservoir intervals (also referred to as seals in this report) to characterize the petrophysical and geo-mechanical properties of these sediments.^5–8^

Depending on their location and formation history, hydrate-bearing reservoirs generally fall into one of three classes, depending on the reservoir’s bounding layers. Class I and Class II reservoirs have the hydrate-bearing zone overlying free-gas- or water-bearing sediments, respectively. Class III reservoirs are bounded by impermeable clay-rich layers above and below the hydrate deposits.^9,10^ The ANS extended-duration gas production test site, where the tested cores in this study were recovered from, has two high-quality B1 and D1 sand reservoir systems in Class I structure, i.e., with free water below. Regardless of the specific class, the interaction between the overburden and the hydrate-bearing zone plays a crucial role in support of gas production since fine-grained overburden sediments with low permeability can act as a seal to prevent or limit the migration of gas and reduce the inflow of water from the overburden into the hydrate-bearing reservoir under depressurization testing.^11,12^ Therefore, it is important to evaluate the producibility of hydrate-bearing reservoirs by considering the geo-mechanical and hydraulic properties of both seal and reservoir sediments in the system.

Methane hydrate requires elevated pressure and reduced temperature conditions to be stable.^13^ To dissociate hydrate and produce gas from the hydrates, multiple extraction methods, including depressurization, thermal stimulation, inhibitor injection, CO_2_ injection, or some combination thereof, have been proposed,^14–19^ considering various types of hydrate occurrences.^20^ Among those, depressurization is believed to be the most promising technique to be used either alone or combined with other production methods.^17,21^ Depressurization can be explained as the drawdown of pore pressure below gas hydrate equilibrium pressures, resulting in hydrate dissociation. However, some consequences of depressurization hamper gas production. Decreases in pore pressure inevitably increase the effective stress on the sediments, leading to potential changes in their mechanical and hydraulic properties. For hydrate-bearing reservoirs, such as at the ANS study site,^8^ destabilization by pressure drawdown was considered. The actual drawdown of pressure at this study site, which was produced continuously for three months, was from 1290 psi (8.89 MPa) to 850 psi (5.86 MPa), i.e., approximately 3.0 MPa bottom hole pressure drop, which was half of the planned depressurization.^6,22^ As has been noted by previous studies, reservoir depressurization will result in an increase in situ effective stress, leading to the reduction of reservoir porosity and a net gain in permeability as gas hydrate dissociates and the reservoir consolidation progresses.^23–25^

This paper focuses on acquiring the mechanical and hydraulic properties of seal and reservoir sediments from B1 and D1 sand reservoir systems at the ANS extended-duration gas production test site^6,7^ to inform the interpretation of the changes in stress, porosity, hydrate saturation, and effective permeability that likely occurred within the tested intervals.

## 2 Specimen Characterizations and Methods

### 2.1 Specimen Characterization

As confirmed by the log data obtained from the test site HYDRATE-01 STW,^4^ the B1 and D1 sands proved to contain significant concentrations of gas hydrate that were suitable for depressurization testing. From the HYDRATE-02 GDW, one sample from the upper seal layers and one sample from the reservoir layers from both the B1 and D1 sands were examined in this study. The specimens, 1P-1A (seal) and 7P-2E (reservoir), are associated with D1 sand, and 13P-3 (seal) and 20P-4 (reservoir) are associated with B1 sand.

The in situ vertical effective stresses σ_v_′ were estimated to be approximately 7 MPa for the specimen 7P-2E from D1 sand reservoir and 9 MPa for the specimen 20P-4 from B1 sand reservoir, using σ_v_′ = ρ*gh*, where the bulk density of overburden sediments is assumed to be ρ = 2000 kg/m^3^, *g* = 9.8 N/kg is the gravity, and *h* is the burial depth of the cores (see Table 1). During depressurization, stable gas and water production was achieved when the pore pressure was decreased by 2.7 MPa and by 3 MPa of bottom hole pressure drop.^6^ Initial plans targeted a pressure drawdown of 7 MPa, which would have increased the vertical effective stress of the D1 sand reservoir to 14 MPa and the B1 sand reservoir to 16 MPa.

**Table 1:** Depths and Index Properties of the Specimens in This Study

	D1 sand (unit D)		B1 sand (unit B)	
core section	1P-1A	7P-2E	13P-3	20P-4
depth [m, TVD]	704.6	723.6	857.4	878.2
core length [cm]	9	34	18	31
porosity, *n* [%]^a^	35.3 (34.4)	30.0 (30.1)	26.5 (27.5)	26.5 (27.2)
*P*-wave velocity, *V*_p_, (m/s)	∼1700	∼2100	∼2000	∼2100
hydrate saturation, *S*_h_ (%)^a^	0.1 (0.5)	32.0 (34.4)	9.3 (11.1)	20.6 (21.7)
soil type	clayey silt, low plasticity	sandy silt	clayey silt, low plasticity	sandy silt
effective particle size, *D*_10_ [μm]	2.7	4.5	0.4	5.3
median particle size, *D*_50_ [μm]	11	32	11	34
liquid limit, LL [%]	34		35	
plastic limit, PL [%]	29		21	
plasticity index, PI [%]	5		14	
specific surface, *S*_s_ [m^2^/g]	38	15	12	4
specific gravity, *G*_s_ [−]	2.68	2.68	2.66	2.72

a Note the tops of the reservoir sands are 710.6 m TVD for D1 sand and 861.5 m TVD for B1 sand. See the project overview^7,8^ for detailed discussions of the GDW depth intervals for the D1 and B1 sand reservoir systems. Values before the parentheses are taken from the on-site analyses by GEOTEK,^26^ and values in parentheses are taken from ref 45.

The porosity and *P*-wave velocity of the samples in this study were taken from the GEOTEK pressure core analysis and transfer system analyses done at the drilling site.^26^ The recovered samples were then depressurized to determine the hydrate saturation from degassing,^27^ followed by vertical splitting into halves for core logging, then shipped to the Georgia Institute of Technology for the mechanical and hydraulic testing.

To avoid the impacts of the vertical splitting of the core samples in the laboratory core measurements, all specimens in this study were remolded using the water pluviation method to simulate natural sedimentation, minimize segregation, and reduce air entrapment during sample preparation. To prepare a dense specimen, targeting the original GEOTEK-based porosities, dry sediments were dropped from a height of 200 mm through a column of deaired water into the sample testing device (e.g., permeability anisotropy cell as described in Section 2.2). Dry mass and sample dimensions were then measured to compute sample porosity, with gentle tapping of the cell walls if further densification of the sample was needed to meet the target initial porosity. The water pluviation method can yield a remolded sample with very similar compression and hydraulic (in the vertical direction) properties as intact sediment samples.^28^ In addition, the sediment grain size distributions were quantified by using the laser diffraction method (Mastersizer 3000E). The fall cone test method (BS 1377) was used to determine the liquid and plastic limits of the seal specimens. The specific surface area of the sediments was determined using the methylene blue method^29^ for particles passing sieve #200 (clay- and silt-size particles). The water displacement method was used to determine the specific gravity of the sediments, following ASTM D854-23. Scanning electron microscopy (SEM) and energy-dispersive X-ray (EDX) analyses were utilized to identify the elemental compositions of the sediments.

### 2.2 Mechanical and Hydraulic Testing

Water retention measurements of the samples were determined using two different methods: the chilled-mirror dew point method (WP4C, ASTM D6836) for fine-grained specimens and the air injection method with a customized cell^30,31^ for coarse-grained specimens. The WP4C Dewpoint PotentiaMeter from METER group is a highly accurate instrument that utilizes the chilled-mirror method to identify the dew point of the sealed chamber and to calculate the relative humidity of the air above a sample in equilibrium. The suction range of the device extends from 0.1 to 300 MPa, allowing its use for fine-grained sediments with high capillarity. The first step of the procedure is to calibrate the device based on instructions in the manufacturer’s manual,^32^ and then prepare the samples with a known water saturation. After placing the sample in the device, the capillary pressure at that specific water saturation was then determined by the device in approximately 20 min using the relationship between water suction potential and the measured relative humidity in the chamber. The sample is then removed from the device and left at room temperature to let it dry and decrease the water saturation. The new water saturation is determined by measuring the mass of water losses, and the previous step for the measurement is then repeated at this lower saturation level until the water saturation of the sample cannot be decreased further due to atmospheric humidity and the water molecules adsorbed on the surface of clay minerals.

The customized water retention cell measures up to the 1.5 MPa air entry pressure of the ceramic disc through which water is expelled during a test (a comprehensive description of the water retention cell is provided in ref 30). The coarse reservoir sediments are tested with the air injection method to have more resolution at low capillary pressures (<0.1 MPa), compared to WP4C, which is mostly suitable for higher pressures. The data collected by both methods are then used to calculate the van Genuchten parameters for each sample^33^

1 $$P_{c}=P_{0}{\left[{\left(\frac{S_{w}-S_{rw}}{1-S_{rw}}\right)}^{-1/m}-1\right]}^{1-m}$$

where the capillary pressure *P*_c_ and the water saturation *S*_w_ are the experimentally measured parameters, while the gas entry pressure *P*_0_, the fitting parameter *m*, and the residual water saturation *S*_rw_ are determined using the least-squares method between the measured and calculated capillary pressure *P*_c_ values at the same saturation level *S*_w_. The gas entry pressure *P*_0_ indicates the air pressure value at which a significant amount of water starts to be drained from the pore space. The fitting parameter *m* describes the shape of the water retention curve, which is governed by the pore size distribution of the specimen. And the residual water saturation *S*_rw_ represents the minimum water saturation that can be reached at the end of the test.

Sediment permeabilities are measured in both the horizontal and the vertical directions using a customized permeability anisotropy cell.^30,31^ The cubic-shaped cell is 35.56 mm (1.4 in) on each side and made of stainless steel walls thick enough to generate less than 10^–6^ strain (i.e., considered as a zero-lateral strain condition) under 35 MPa stress. The chamber has one inlet and two outlets in both horizontal and vertical directions, enabling measurements of both vertical permeability, *k*_v_, and horizontal permeability, *k*_h_, for the same sample. Each outlet is fitted with two isolated porous stones, an inner circular one within an outer square one, forming the ‘double-ring’ pattern (ASTM D3385 and ASTM D5093). The outer outlet is designed to collect the preferential flow between the cell wall and the sample, while the inner outlet collects water that passes only through the sediments. It is only the flow through the inner outlet that is used for permeability measurement. Based on Darcy’s law, the permeability *k* in each direction is determined by applying a pressure difference Δ*P* across the sample using an ISCO pump at the inlet and outlets of the cell and measuring the corresponding flux *Q* through the inner ring

2 $$k=\frac{QL}{\Delta PA_{in}}\mu$$

where *L* is the height/width of the sample for vertical/horizontal flow; *A*_in_ is the area of the inner circular porous stone for flow through the sample; and μ is fluid viscosity. The hydraulic gradient *i* across the sample depends on the applied differential pressure Δ*P* as *i* = Δ*P*/(*L*γ_w_), where γ_w_ is the unit weight of water. All horizontal injection and effluent ports are closed when measuring the vertical permeability. All of the vertical injection and effluent ports are closed for horizontal permeability measurements. Each measurement uses about 2–5 pore volumes of fluid to reach a steady-state flux through the tested sample.

Both horizontal and vertical permeabilities are measured at the end of consolidation at different stresses in the customized permeameter. For hydrate-free cores, D1 sand (unit D) is stepwise loaded under stresses of 0.01, 0.1, 0.5, 1, 3, 5, and 7 MPa (in situ stress), followed by unloading to 5, 3, and 1 MPa. B1 sand (unit B) is loaded to 0.01, 0.1, 0.5, 1, 3, 5, 7, and 9 MPa, followed by unloading to 5, 3, and 1 MPa. For the two hydrate-bearing cores (7P-2E and 20P-4), each core is loaded stepwise as the hydrate-free core until its in situ stress; the hydrate is then dissociated under this stress, followed by continued loading of the post-hydrate-dissociation cores to 12 MPa for core 7P-2D and 16 MPa for core 20P-4. The consolidation under each loading condition is considered to be completed when no detectable settlement is observed.

The hydrate-bearing properties of the reservoir samples are quantified using tetrahydrofuran (THF) as the hydrate-forming proxy for methane. Three main reasons for using THF in this study include (1) THF hydrate is stoichiometric, so hydrate saturation can be accurately controlled (note that the cage occupancy of methane hydrate can vary). (2) THF is soluble in water and can form hydrates in fully water-saturated specimens, while the saturation process in specimens with hydrate formed at unsaturated conditions can be difficult and result in hydrate dissolution and/or dissociation. And (3) THF hydrate forms at atmospheric pressure, which significantly simplifies the testing conditions to allow accurate control of pressure and stresses when measuring geo-mechanical and hydraulic properties of hydrate-bearing sediments. Laboratory synthesis of hydrate-bearing sediments using THF hydrate allows high reproducibility, especially for studying the influence of hydrate saturation in the pore space on the sediment’s geo-mechanical and geo-physical properties.^34^

Despite the differences between THF and CH_4_ molecules in terms of size and polarity, as well as the different cage structures of the formed hydrate (i.e., methane hydrate: Structure I, THF hydrate: Structure II), THF hydrate has similar physical (e.g., density) and mechanical (e.g., Young’s modulus, bulk compressibility, and shear/compressional wave velocities) properties as those of methane hydrate.^35,36^ There have been numerous laboratory studies deploying THF hydrate to understand the impacts of hydrate saturation on the geo-mechanical,^37–39^ geo-physical,^40,41^ and hydraulic^42,43^ properties of hydrate-bearing sediments. However, very limited studies directly compare the properties of THF-hydrate-bearing and natural methane-hydrate-bearing sediments, i.e., pressure cores^28^ showed that THF-hydrate-bearing sediments can provide meaningful geo-mechanical properties complementary to pressure core testing, yet with higher permeability than pressure core measurements. Admittedly, the suitability to replace methane hydrate with THF hydrate still requires further research.

The required proportions of water and THF are mixed to reach the desired hydrate saturation level in the pore space.^34^ Specifically, 3.89 wt % THF in the THF-H_2_O solution to form *S*_h_ = 20.6%, and 6.05 wt % THF in the THF-H_2_O solution to form *S*_h_ = 32%. After hydrate formation, only the extra water phase coexists with the THF hydrate in the pores in this study. The samples for both water retention and permeability tests are prepared using pluviation into the water-THF mixture. The samples are then kept in an environmental chamber (Associated Environmental Systems, LH-10), set at −10 °C, to initiate hydrate formation while using a thermocouple to monitor the temperature. After observing a thermal spike at a temperature above 0 °C, indicating hydrate formation due to the exothermic response of THF hydrate crystallization, the environmental chamber temperature is then raised to 1 °C and kept at this temperature for 24 h to ensure continued hydrate formation with ice formation. Note also that reported hydrate saturations are at the cores’ initial no stress state (not under in situ stress), which may increase upon loading as the pore volume decreases.

## 3 Experimental Results

### 3.1 Index Properties

Index properties of the specimens are summarized in Table 1, including both the in situ and lab characterization. The sediment grain size distributions are shown in Figure 1a. According to the Wentworth grain size classification^44^ (sand: 62.5 μm–2 mm, silt: 4–62.5 μm, and clay: < 4 μm), the seal specimens, 1P-1A and 13P-3, are identified as clayey silt. However, compared to 1P-1A, 13P-3 has a wider grain size distribution with a lower *D*_10_ = 0.4 μm (note that *D*_10_ approximates the pore size in the specimen and is typically called the effective particle size used in estimating soil permeability) and a higher percentage of sand-size particles (20%). The two reservoir specimens, 7P-2E and 20P-4, can be classified as sandy silt with significant similarities in grain size distributions and median particle sizes (*D*_50_). The fall cone test results suggest that both seal specimens are clayey materials with low plasticity. The specific surface area data imply the possible presence of kaolinite in all four tested specimens due to the low surface area measurements (note: surface areas for typical clays—kaolinite: ∼10–30 m^2^/g, Illite: ∼65–100 m^2^/g, chlorite: ∼70–120 m^2^/g, and montmorillonite: ∼600–1000 m^2^/g).

**Figure 1:**
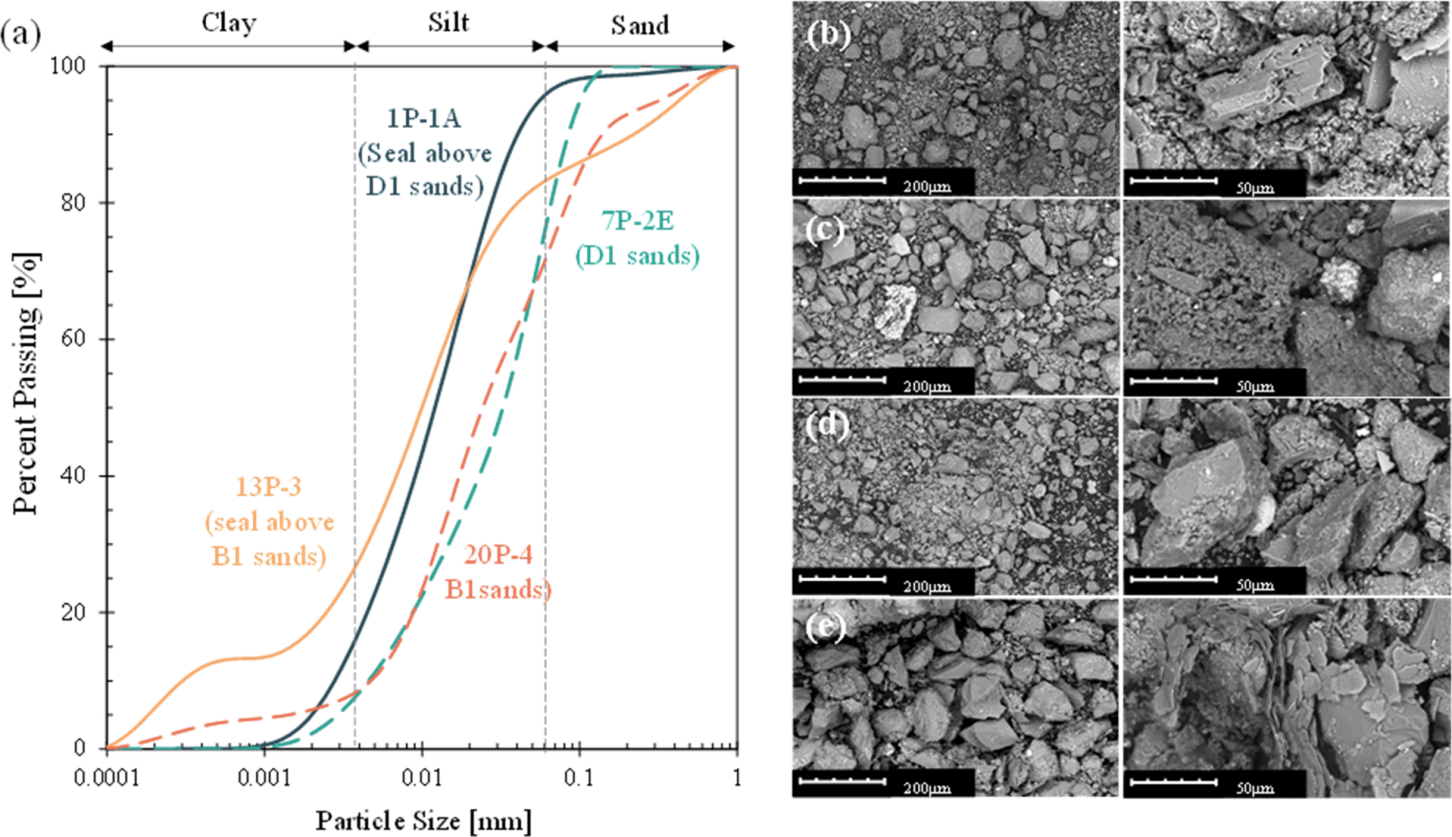
Sediment particle analysis. (a) Grain size distributions of the four tested samples in this study. Scanning electron microscope images of the seal and the reservoir specimens: (b) seal above the D1 sand 1P-1A, (c) D1 sand 7P-2E, (d) seal above the B1 sands 13P-3, and (e) B1 sands 20P-4.

SEM photographs in Figure 1b–e show the presence of rectangular prism-shaped precipitates and light-colored, circular minerals in sediments. The clayey silt specimens include plate-like structures, while the sandy silt specimens mostly consist of angular particles. Under the same scale, the size differences between the seal and reservoir sediments are seen clearly in the first column of Figure 1b–e. To determine the elemental composition of the materials, EDX analysis was used. The results of the tested sediments are listed in Figure 2. Each set of test results is shown with unique color coding in Figure 2, and data are given in terms of the fraction of the highest peak of that test at varying energy levels in kiloelectronvolts. The tested precipitate particle (green line) shows the existence of calcium and oxygen in high amounts, indicating the presence of calcium carbonate (CaCO_3_). The presence of calcium precipitates indicates the chemical weathering of limestone bedrock by rivers and groundwater flows.^46^ The light-colored, ball-shaped particles (blue line) are mostly composed of iron and sulfur, which is a sign of iron pyrite (FeS_2_) in the sediments. The formation of iron pyrite takes place with the reaction of iron from muddy sediments and sulfur from the microbial reduction of seawater sulfate.^47^ Finally, the soil particle (orange line) is made of oxygen, aluminum, and silicon, as expected for a clay mineral.

**Figure 2:**
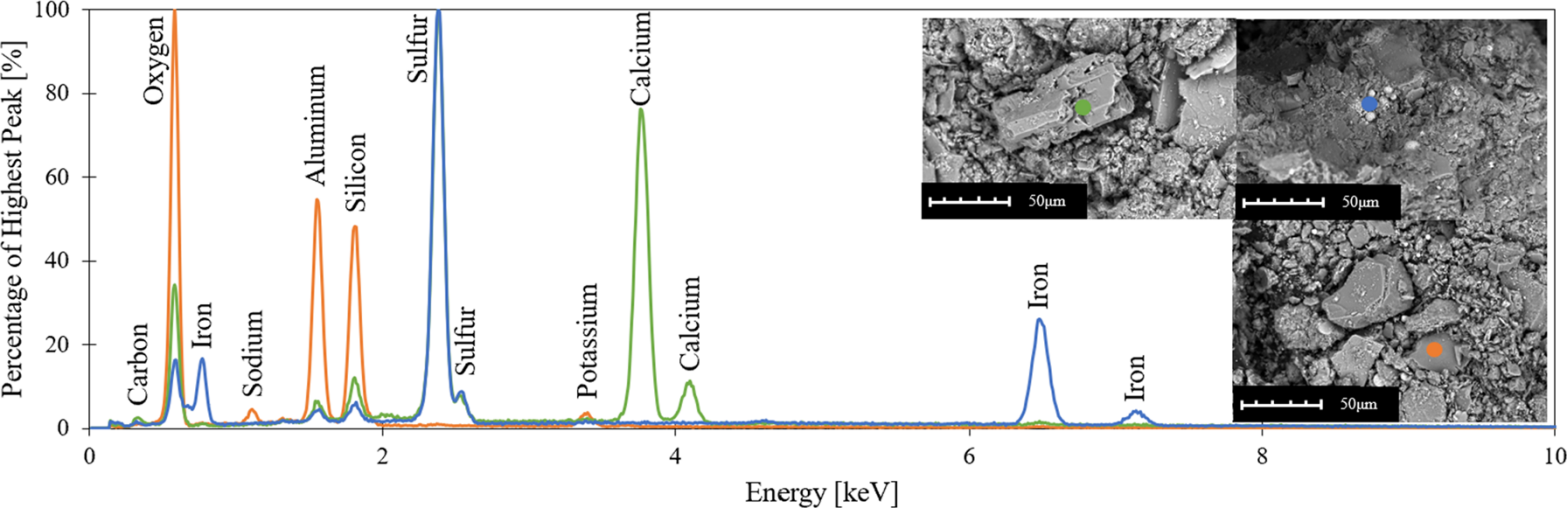
EDX analysis results, highlighting the presence of calcium carbonate (CaCO_3_) and iron pyrite (FeS_2_) in the sediments.

### 3.2 Consolidation

Sample consolidation [under vertical stress up to 7 MPa for D1 sand (unit D) and 9 MPa for B1 sand (unit B)] is performed using the customized permeameter. Results for the four specimens with no hydrate are shown in Figure 3a. Each specimen is prepared at a starting void ratio of *e* = 0.72, with the purpose of reaching the in situ void ratio range of *e* = 0.35–0.43 at their in situ stress levels. The seal specimens show a higher compressibility with a compression index *C*_c_ of 0.28 for the sediments directly overlying the D1 sand and 0.26 for the sediments overlying the B1 sands, compared to the hydrate-free reservoir sediments all with a compression index of 0.24. Even though the specimens are remolded and have not experienced a preconsolidation stress, the data points prior to approximately σ_v_′ = 1 MPa have a slope different than the virgin slope, possibly due to the initial seating effects of the permeameter. The compression indices for all specimens are calculated by using the slope between σ_v_′ = 1 MPa and the final in situ stress.

**Figure 3:**
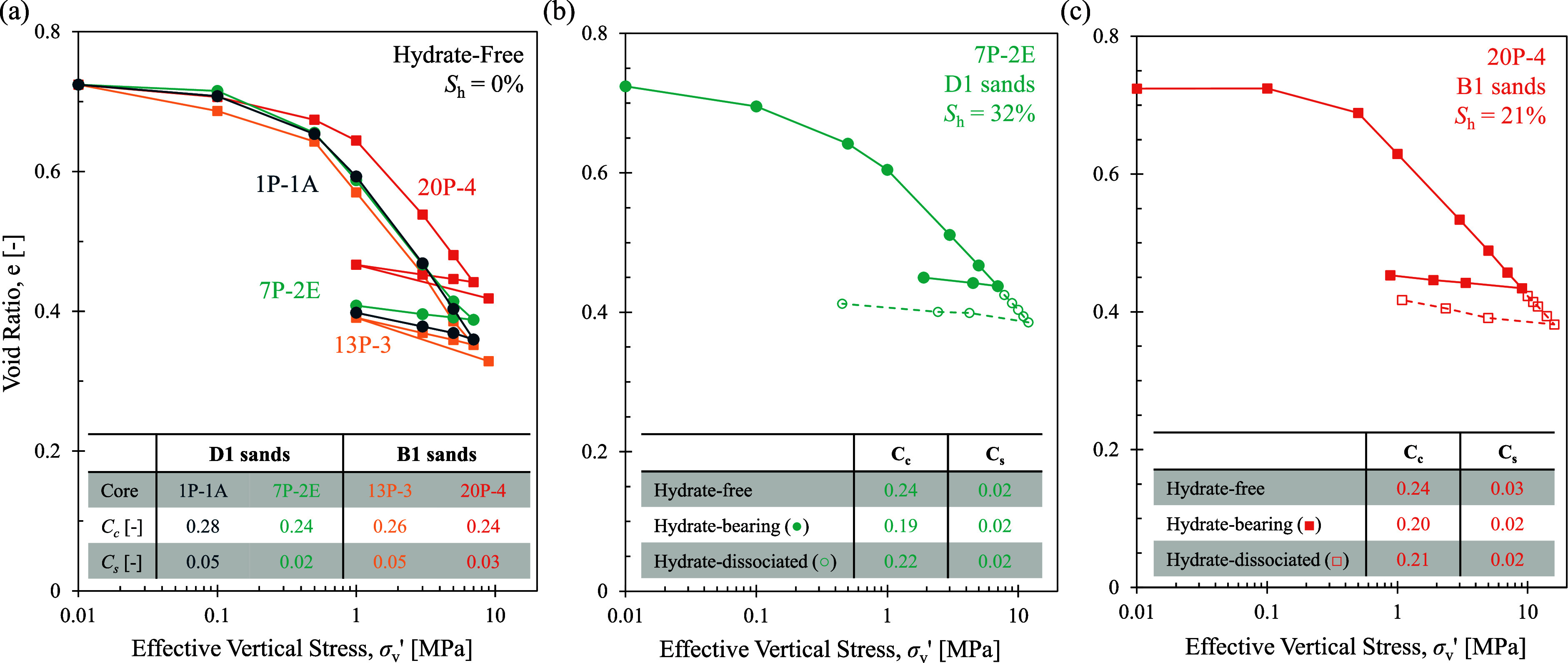
Consolidation results for (a) remolded samples with no THF hydrate, (b) D1 sand (unit D) reservoir sample (7P-2E) before and after hydrate dissociation, and (c) B1 sand (unit B) reservoir sample (20P-4) before and after hydrate dissociation.

The consolidation test for the hydrate-bearing specimens was conducted under two conditions: before and after hydrate dissociation. After reaching the in situ stress with the hydrate-bearing specimen, the environmental chamber is turned off to let the specimen warm so the hydrate will dissociate at the in situ stress (i.e., 7 MPa for core 7P-2E and 9 MPa for core 20P-4) level. Then, the dissociated specimen is loaded to 12 MPa for the D1 sand (unit D) and 16 MPa for the B1 sand, corresponding to the in situ stress after a depressurization of 5 MPa in the D1 and 7 MPa in the B1. The compressibility of the dissociated specimens is determined by using the slope of the data in open markers (note: solid markers in Figure 3 are data for specimens with hydrate, and open markers are post-hydrate-dissociation data). Figure 3b and c show the consolidation data for each unit’s hydrate-bearing and post-dissociation samples. The compression index of the hydrate-bearing specimen is *C*_c_ = 0.19 and 0.20 for D sand (unit D) and B sand (unit B), respectively, even though the D sand (unit D) has 11% higher hydrate saturation than the B sand (unit B). These results suggest that even a low hydrate saturation (21%, B sand) is enough to reduce the sediment compressibility measurably, but that between *S*_h_ values ∼20–35%, compressibility is not strongly correlated with hydrate saturation.^48^ The hydrate dissociation increases the compression index by approximately 0.03 for the D1 sand and 0.01 for the B1 sand; however, the compression indices of the hydrate-free specimens are still greater at *C*_c_ = 0.24. There is no significant difference in the swelling indices *C*_s_ between sediment types or hydrate saturations in this study.

### 3.3 Water Retention Curves

Figure 4 summarizes the water retention data for all tested samples. The hydrate-bearing samples are noted with their determined *S*_h_, which represents the THF hydrate presence that occurs as a pore-filling material. The hydrate-bearing samples were fully water-saturated at the beginning of the water retention test. Even though the B1 sand seal specimen (13P-3) has a wider grain size distribution than that of the D1 sand seal specimen (1P-1A), they both show a similar capillary behavior. The air injection method for the hydrate-free reservoir samples (7P-2E and 20P-4) was applied for the pressure range between 20 and 700 kPa, above which no additional drainage can be measured within an accuracy of 0.01 g.

**Figure 4:**
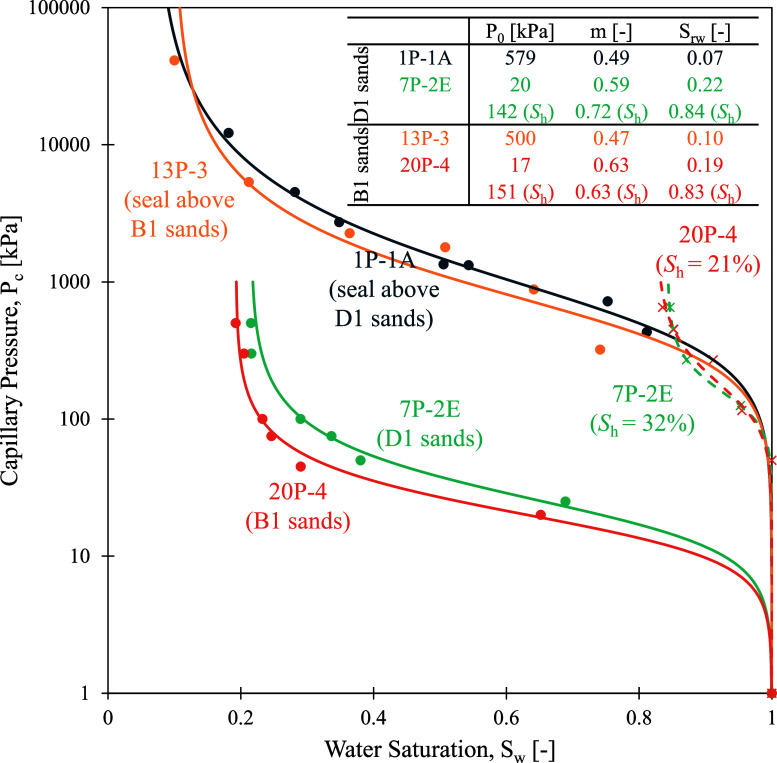
Water retention test data (scattered points) fitted using the van Genuchten model for all the specimens (solid curves for hydrate-free samples, dashed for hydrate-bearing reservoir sediment). The inset table shows the determined van Genuchten parameters for each specimen.

The hydrate-bearing reservoir samples are tested within the same 20–700 kPa pressure range. The gas entry pressure for the hydrate-bearing reservoir sediments is significantly greater than that for the hydrate-free sediments. Similarly, the residual water saturation *S*_rw_ of the hydrate-bearing reservoir samples is greater than that in the hydrate-free samples, as hydrate blocks and/or narrow the flow pathways for water drainage.

With increasing fine percentage in the specimen, the gas entry pressure increases significantly from ∼20 kPa for reservoir specimens to ∼580 kPa for seal specimens. The presence of hydrate in the pore space also changes the capillary behavior of the reservoir specimens by increasing their air entry pressure and the residual saturation. The fitting parameter *m* in eq 1 is correlated to the width of the pore size distribution of the specimen. The lowest *m* values in the seal specimens indicate a wider distribution of pore sizes, compared to that in the reservoir sample with and without hydrate. Hydrate formation increases the *m* value, resulting in a more uniform pore size distribution in the reservoir specimen, compared to the wider distribution in the hydrate-free specimens.

### 3.4 Horizontal and Vertical Permeability

Measured vertical and horizontal permeability in tested specimens is shown in Figure 5. The starting permeability (at σ_v_′ = 10 kPa) in all four hydrate-free sediment specimens is higher in the horizontal direction *k*_h_, than in the vertical direction *k*_v_. This may be due to water pluviation-induced horizontal layers (coarser grains settle faster than finer ones), so that the horizontal flow tends to follow more permeable layers, but the vertical flow must cross through low-permeability (i.e., fine-grained) layers. Both *k*_v_ and *k*_h_ decrease with increasing vertical effective stress σ_v_′, and the stress-induced permeability reduction is greater in the horizontal direction than in the vertical direction, as the specimen is compressed vertically in the oedometer during loading. Note that some cores show a slight increase in permeability with increased stress when the stress level is low, i.e., 0.01–0.5 MPa. This may be caused by multiple sources of error, such as gas bubbles that existed at the beginning of the test were flushed out later, poor contact between the core and boundary porous stone (i.e., seating effect), or clayey particle fabric change during the fluid circulation for permeability measurement. However, they all decrease with increasing stress after 0.5 MPa vertical effective stress. On the other hand, these measured permeability at low stresses (<0.5 MPa) are not relevant to field behavior, nor are they used in any of the following analyses.

**Figure 5:**
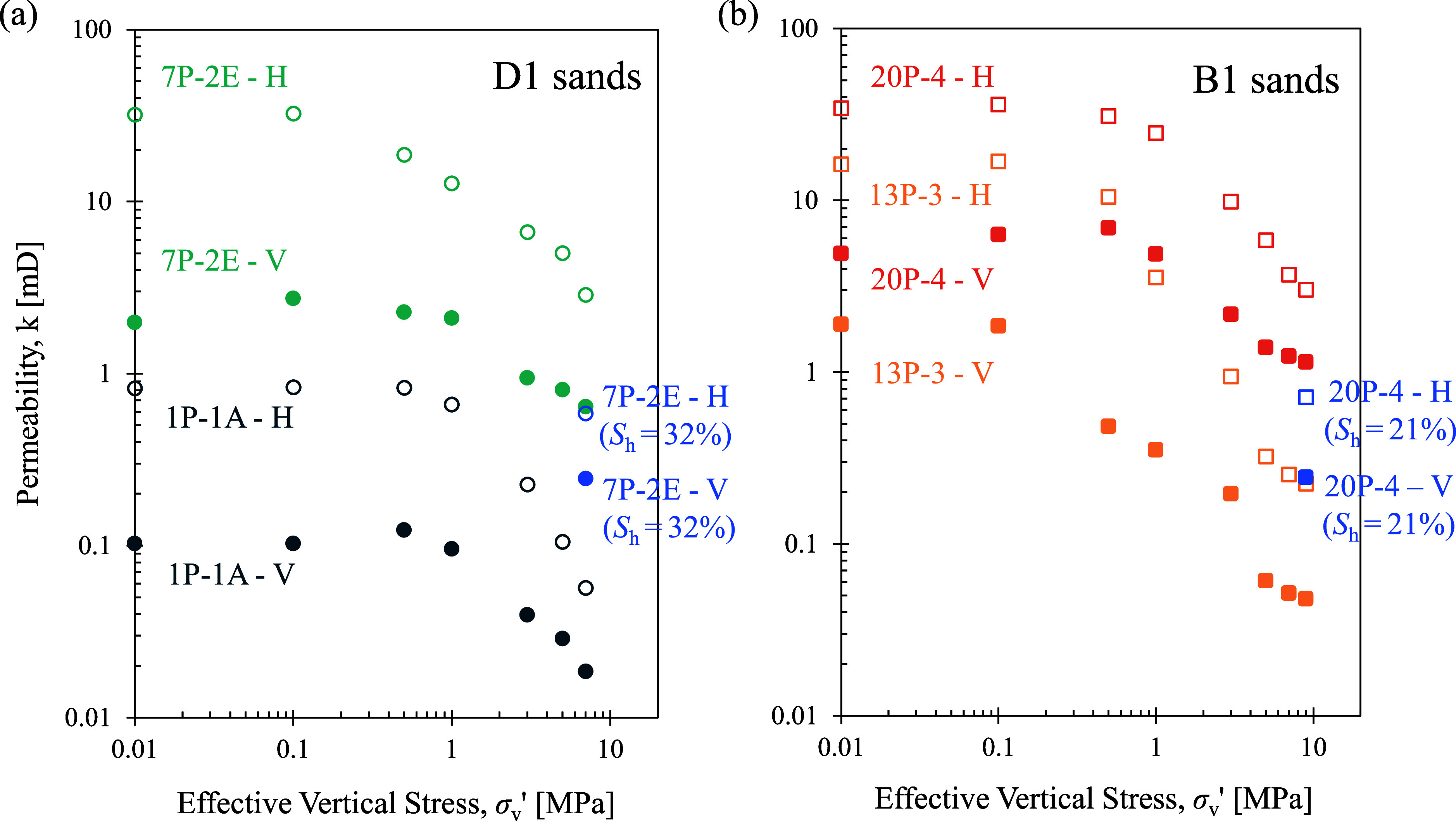
Permeability measurements in vertical (−V, solid symbols) and horizontal (−H, open symbols) directions in all tested samples. Measurements made in hydrate-bearing reservoir sediment are labeled with the hydrate saturation, *S*_h_.

For the D1 sand (unit D), the hydrate-free specimen (7P-2E) has a permeability of 0.6 mD in the vertical direction and 2.9 mD in the horizontal direction at in situ effective vertical stress of σ_v_′ = 7 MPa. This is at least an order of magnitude higher than the associated seal specimen (1P-1A), characterized by 0.02 mD in the vertical direction and 0.06 mD in the horizontal direction. On the other hand, the B1 sand sample (20P-4) exhibited about doubled permeability values compared to its associated seal sample (13P-3) at low-stress levels. However, with increasing effective vertical stress, the B1 sand specimen (20P-4) reaches a vertical permeability of 1.1 mD and a horizontal permeability of 3.0 mD at σ_v_′ = 9 MPa. The permeability of the seal specimen (13P-3) decreases to 0.05 mD in the vertical direction and 0.22 mD in the horizontal direction when the in situ stress conditions are reached. The predicted vertical permeability for this core is 0.0005–0.03 mD.^49^

To minimize hydrate dissolution due to water circulation for permeability measurement, the permeability of hydrate-bearing samples is measured only at their in situ effective vertical stress levels. The circulation fluid for permeability measurement uses the THF–water mixture with the same stoichiometric ratio for hydrate formation to ensure unchanged hydrate saturation during testing. The hydrate-bearing sediments from the D1 sand (unit D) with hydrate saturation *S*_h_ = 32% have a vertical permeability of 0.2 mD and a horizontal permeability of 0.6 mD at σ_v_′ = 7 MPa. The hydrate in the pore space impacts the horizontal permeability more drastically than the vertical permeability. The presence of 21% hydrate in the reservoir specimen of the B1 sand (unit B) decreases the vertical permeability from 1.1 mD to 0.3 mD and the horizontal permeability from 3.0 mD to 0.7 mD. After hydrate dissociation, the permeability of the samples returned to the hydrate-free values for the reservoir samples in both a vertical and horizontal direction. Hydrate formation and dissociation can cause volume changes in the pore space, particularly when hydrate saturation is high (*S*_h_ > 70%).^48^ The low hydrate saturation of the sediments in this study (*S*_h_ = 21% in 20P-4 and 32% in 7P-2E) does not lead to significant volume change during hydrate dissociation (void ratio reduction is less than 0.01, see Figure 3b,c).

## 4 Analyses and Discussion

### 4.1 Permeability Anisotropy

Natural sedimentary reservoirs tend to exhibit anisotropic permeability properties due to macro- and microstratification of the reservoir.^50,51^ In addition, the preferential alignment of nonspherical particles during deposition, sedimentation, or flow currents^52^ can increase the anisotropy of a given system. Platy particles, such as clay particles, tend to align with the large axis oriented horizontally and the small axis vertically for stability. For hydrate-bearing sediments, depending on hydrate saturation and the pore structure, hydrate formation can occur in different forms, such as pore-filling or grain-displacing.^53^ The spatial distributions of hydrate, pore shape, and throat sizes impact the permeability in different directions.^54–56^

Collected data on permeability anisotropy (*k*_h_/*k*_v_, the ratio of the horizontal over the vertical permeability) at different stress conditions in this study are shown in Figure 6. With water pluviation, the remolded samples likely have a graded bed where larger particles settle faster than finer ones, resulting in high permeability anisotropy ratios at low vertical effective stress. With increasing vertical stress, the decrease in the horizontal permeability exceeds that in the vertical direction, resulting in lower anisotropy ratios that reach approximately a ratio of *k*_h_/*k*_v_ = 3.0–5.0 for the hydrate-free sediments at the in situ effective vertical stress of 7–9 MPa. The THF hydrate-bearing sediments have an anisotropy ratio of *k*_h_/*k*_v_ = 2.4 for the D1 sand and *k*_h_/*k*_v_ = 3.0 for the B1 sand. A faster decrease in the horizontal permeability than the vertical permeability during loading stems from the more rapid reduction in the vertical cross-sectional pore-space area for horizontal flow than in the horizontal cross-sectional area for vertical flow. Admittedly, these measurements reflect the sediment fabric from gravitational settlement in a quiescent water environment and do not necessarily capture the in situ fabric out of the pelagic processes, nor the interbedded layers at a larger scale.

**Figure 6:**
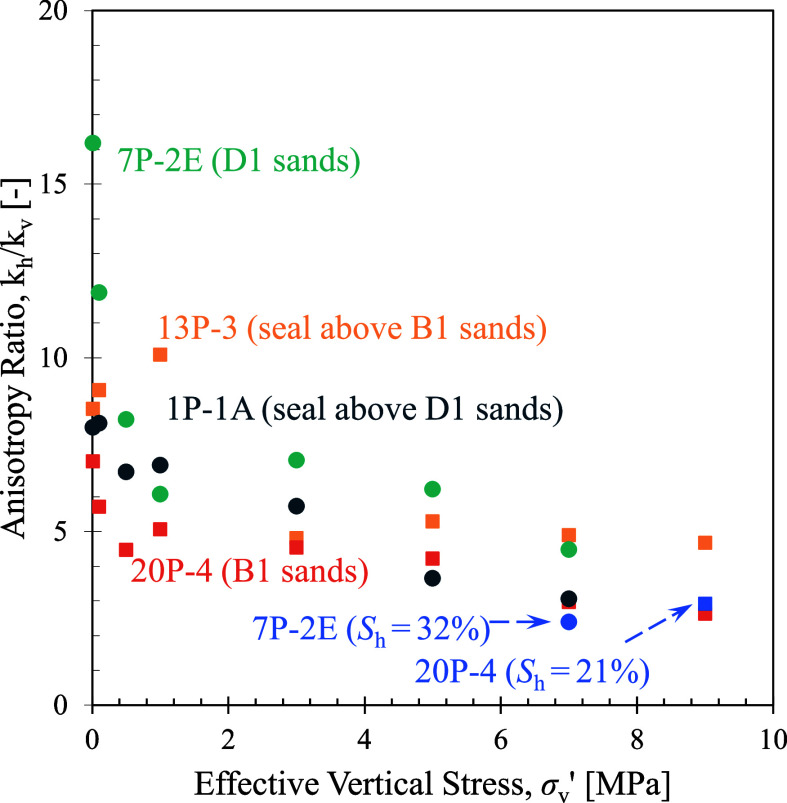
Permeability anisotropy ratio for the hydrate-free and hydrate-bearing sediments as a function of effective vertical stress.

### 4.2 Relative Permeability

The van Genuchten model can be used to describe the relative permeability to water (*k*_rw_) and to gas (*k*_rg_) at different water saturations,^57^ using the same fitting parameter *m* and residual water saturation *S*_*rw*_ determined from the water retention curves:

3 $$\begin{array}{l} k_{rw}={\mathrm{S̅}}^{0.5}{\left[1-{\left(1-{\mathrm{S̅}}^{1/m}\right)}^{m}\right]}^{2}\\ k_{rg}={\left(1-\mathrm{S̅}\right)}^{0.5}{\left[1-{\mathrm{S̅}}^{1/m}\right]}^{2m} \end{array}$$

where the effective water saturation is $\mathrm{S̅}=\left(S_{w}-S_{rw}\right)/\left(1-S_{rw}\right)$ and for the hydrate-bearing case, *S*_w_ + *S*_g_ + *S*_h_ = 1. The relative permeability curves for the hydrate-free and hydrate-bearing sediments are shown in Figure 7a,b, respectively. The solid curves represent the relative water permeability, while the dashed curves represent the relative gas permeability at corresponding water saturation levels. The lower *m* value implies a lower water permeability but a higher gas permeability at the same saturation,^58^ and this trend can be seen between the reservoir and seal sediments in Figure 7a. The relative permeability to water dominates when water saturation is above 85% in seal sediments and above 80% in the reservoir sediments. On the other hand, the saturation range for the water permeability is limited in the hydrate-bearing sediments, as seen in Figure 7b. The clogging of the pore throats due to the presence of hydrate prevents water drainage, resulting in higher residual water saturation and the dominance of gas permeability even at relatively high water saturations.

**Figure 7:**
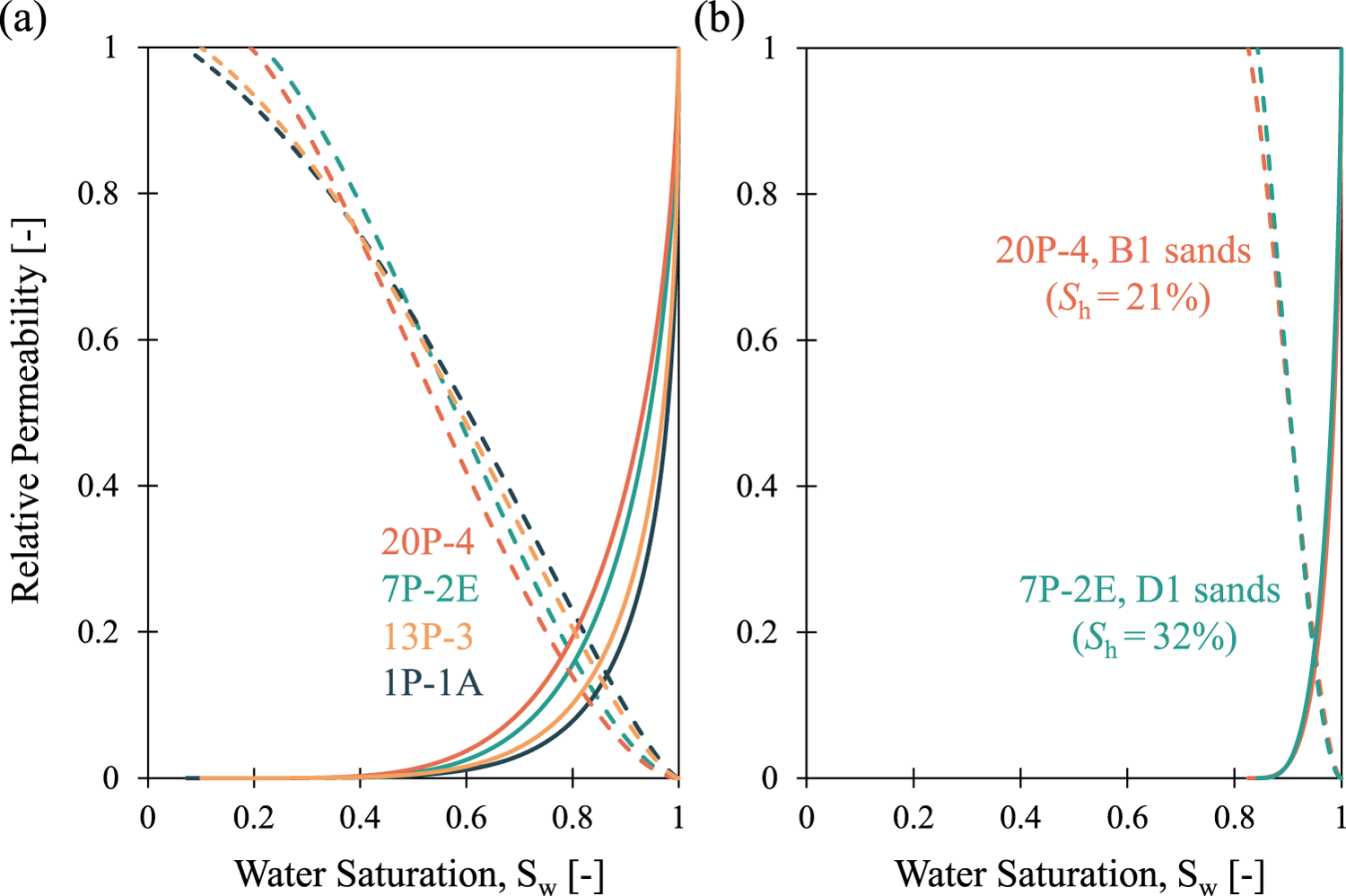
Relative permeability to water (solid curves) and gas (dashed curves) of (a) hydrate-free sediments and (b) hydrate-bearing reservoir sediments.

### 4.3 Reservoir Volume and Permeability Responses during Gas Production by Depressurization

Depressurization to produce gas from hydrate results in an increase in the effective stress on the sediments. Consequently, reservoir deformation becomes inevitable. With known in situ effective vertical stress σ_in situ_ and the sediment’s compression (*C*_c_) and swelling (*C*_s_) indices, we can calculate the change in void ratio Δ*e* due to depressurization Δ*P*

4 $$\Delta e=C_{c/s}{\;\log}_{10}\left(\frac{\sigma_{in‐situ}+\Delta P}{\sigma_{in‐situ}}\right)$$

Then, with the reservoir thickness *H*_0_ and the in situ void ratio *e*_0_, total settlement Δ*H* can be determined as

5 $$\Delta H=H_{0}\left(\frac{\Delta e}{1+e_{0}}\right)$$

Given that the D1 sand (unit D) has a reservoir thickness *H*_0_ of 18 m, an in situ effective vertical stress σ_in situ_ of 7 MPa, an in situ void ratio *e*_0_ of 0.43, a compression index *C*_c_ of 0.22 (after hydrate dissociation), and a swelling index of *C*_s_ of 0.02 (after hydrate dissociation), the reduction in void ratio and total settlement due to depressurization can be calculated (Figure 8). For the B1 sand, the reservoir has a thickness *H*_0_ of 21 m, an in situ effective vertical stress σ_in situ_ of 9 MPa, an in situ void ratio *e*_0_ of 0.37, a compression index *C*_c_ of 0.21 (after hydrate dissociation), and a swelling index of *C*_s_ of 0.02 (after hydrate dissociation).

**Figure 8:**
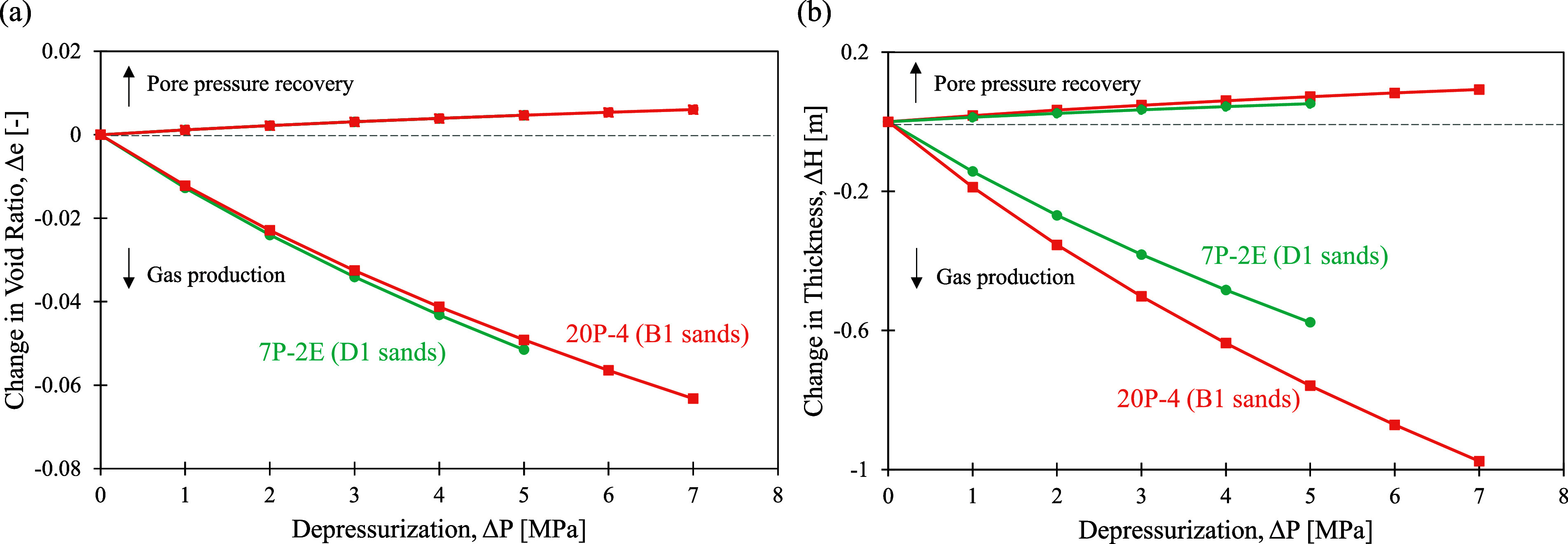
(a) Change in void ratio and (b) change in thickness in the hydrate-dissociated reservoir layers of each unit during depressurization. Negative values represent compression, while positive values represent swelling. For this calculation, the reservoir sand thicknesses are taken to be 18 m (58 ft) for the D1 sand and 21 m (66 ft) for the B1 sand.

Negative values reflect compression during gas production, and positive values for swelling during pore pressure recovery. According to the idealized production plan, the lowest possible pore pressure after drawdown is 2 MPa. For the D1 sand, drawing pore pressure from approximately 7 to 2 MPa in the 18 m-thick reservoir would decrease the thickness by 0.7 m, while for the B1 sand, a pore pressure change from 9 to 2 MPa in the 21 m-thick reservoir would result in a thickness decrease of 1.0 m. After gas production efforts have been completed and the pore pressure recovers, the reservoir swells according to the swelling index *C*_s_, and the effective stress decreases. However, in the reservoir sediment, *C*_s_ is an order of magnitude smaller than *C*_c_, so the recovered deformation is much smaller than the sediment compaction, resulting in a permanent reservoir settlement. For example, given a 3 MPa pressure drawdown, it is expected to have a settlement of 0.4 and 0.5 m in D1 and B1 sands at the end of gas production and a bounce back of 0.04 and 0.05 m in D1 and B1 sands after pore pressure recovery. Note that these deformation calculations are theoretical and estimate the final reservoir thickness changes after complete hydrate dissociation and full pore pressure recovery. These estimations do not capture the time-lapse deformation, which is affected by the rates of depressurization, hydrate dissociation, and pore pressure recovery. In addition, the drawdown of 440 psi (i.e., ∼3 MPa) induced about 6 mm deformation in the well cement in B1 sands according to measurements from the embedded distributed strain sensing cable.^59^ Assuming linear elasticity for first-order estimations herein, the sediment stiffness is estimated to be *E*_s_ = Δσ/ε = Δσ/[Δ*e*/(1 + *e*_0_)] = 3 MPa/[0.033/(1 + 0.37)] = ∼124.6 MPa, while the cement stiffness is approximately *E*_c_ = ∼5–10 GPa. Therefore, the deformation in the reservoir should be about 40–80 times that in the cement well for force equilibrium. Note that the theoretical final settlement in the reservoir, (0.5–0.05) m = 0.45 m, is about 75 times that measured in the cement well (0.006 m).

During depressurization, two competing factors govern the modification of the in situ sediment permeability: increased effective stress squeezes the pore space, decreasing sediment permeability, while the loss of hydrate crystals opens up the pore space, increasing sediment permeability. Using a power trendline for the virgin curve portion (between σ_v_′ = 1 MPa and in situ stress) of the permeability profile in Figure 5, the horizontal permeability in hydrate-free sediments *k*_HF_ at any stress σ_v_′ for the D1 and B1 reservoirs can be described as

6 $$k_{HF,D}=k_{HF,D}^{1\;MPa}\left[{\left(\frac{\sigma_{v}^{\prime }}{1\;MPa}\right)}^{-0.71}\right]$$

7 $$k_{HF,B}=k_{HF,B}^{1\;MPa}\left[{\left(\frac{\sigma_{v}^{\prime }}{1\;MPa}\right)}^{-0.97}\right]$$

where *k*_HF_^1 MPa^ is the hydrate-free horizontal permeability at σ_v_′ = 1 MPa and the subscripts B and D refer to the geologic unit. On the other hand, the impact of hydrate saturation, *S*_h_, on permeability at a given porosity can be predicted as^60^

8 $$k_{HF}=k_{HB}\left[\frac{{\left(1+2S_{h}\right)}^{2}}{{\left(1-S_{h}\right)}^{3}}\right]$$

where *k*_HF_ is the hydrate-free permeability and *k*_HB_ is the hydrate-bearing permeability. Since these two factors are competing to change permeability during depressurization, a line for zero-horizontal-permeability-change can be determined when the changes in horizontal permeability due to effective stress increase and hydrate dissociation are equal.

As shown in Figure 9a, the zero horizontal permeability change lines for each unit are shown with respect to the initial hydrate saturation and their in situ stresses. The region above the lines represents an overall gain in the final permeability after depressurization compared to the initial hydrate-bearing permeability, as the permeability increase due to hydrate dissociation dominates the stress-induced permeability decrease. Conversely, the region below the lines represents a lower permeability after depressurization than that of the initial hydrate-bearing permeability. As suggested in Figure 9a, both units are expected to experience an overall increase in the horizontal permeability in the reservoir at the end of the depressurization (i.e., postgas production) compared to that before the gas production. This is because the reservoir sediments at this site are less compressible, so the permeability reduction by stress increase is not as significant as the hydrate-loss-induced permeability increase. However, at sites where sediments are more compressible, stress increase-induced permeability reduction may exceed hydrate loss-caused permeability increase, resulting in an overall permeability decrease in the reservoir at the end of gas production. Based on the impacts of hydrate dissociation (eq 8) and stress change (eqs 6 and 7 for the B1 and D1 sands) on horizontal permeability, Figure 9b shows the permeability ratio before and after gas production by depressurization at different depressurization values.

**Figure 9:**
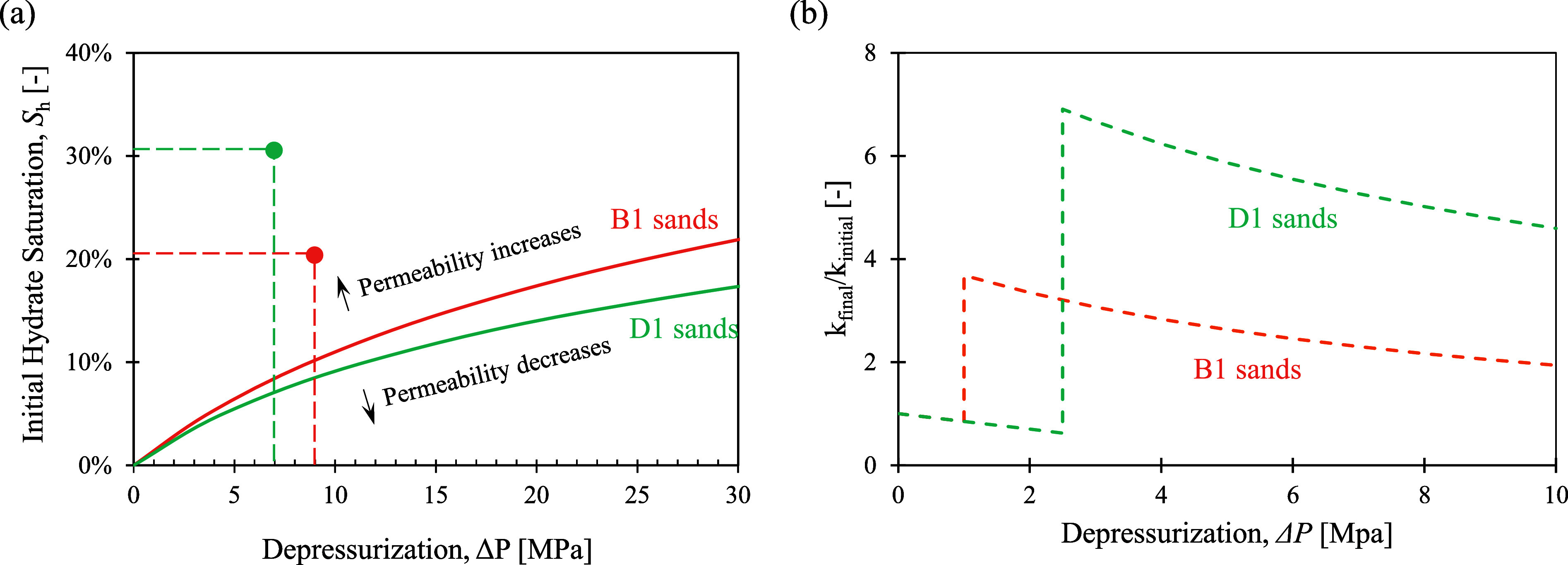
Horizontal permeability changes during gas production. (a) The zero-horizontal-permeability-change lines for the B1 and D1 sand reservoirs. The dots represent the initial hydrate saturation and the potential depressurization pressure in the B1 and D1 sand reservoirs. (b) The normalized reduction in horizontal permeability with increased depressurization pressure.

### 4.4 Limitations in the Methods Used in This Study

The two primary limitations of the methods employed in this study are associated with sample remolding and the use of a THF hydrate as a proxy.

The remolded samples in this study used water pluviation, which can potentially result in vertical grading, i.e., larger particles settle faster than fines through water. This is evidenced by the initial permeability anisotropy in all tested samples, which is relatively high *k*_h_/*k*_v_ = ∼6–16. It is worth highlighting that water pluviation does not mimic actual deposition in situ, where water currents, bioturbation, compaction, and chemical processes alter sediment fabric and porosity. Unfortunately, the permeability (and permeability anisotropy) in the samples studied here has not been measured prior to splitting the cores into halves. At in situ stresses, measured permeability anisotropy decreases to *k*_h_/*k*_v_ = 5 in core 7P-2E from D1 sands at 7 MPa, and to *k*_h_/*k*_v_ = 3 in core 20P-4 from B1 sands at 9 MPa. As a reference, the permeability anisotropy in cores 16P-1e and 17P-5f from B1 sands using NMR in pressures are *k*_h_/*k*_v_ = 5 and 0.4, respectively.^61^ Admittedly, to what extent the fabric in water pluviated samples replicates their in situ conditions is unknown and requires further investigation.

Figure 10 summarizes the measured vertical permeability in this study and various other cores^49,62^ from the same testing site. Measured permeability is not directly related to porosity *n* or media grain size *D*_50_ alone for different cores (Figure 10a,b). According to Kozeny^63^ and Carman,^64^ the permeability of a porous medium is proportional to the square of the characteristic particle diameter *d* ^2^ and the cube of porosity *n* ^3^. Figure 10c shows that the measured permeability correlates with (*D*_50_^2^ × *n* ^3^) well for all cores with reported permeability values from the Alaska testing site in the following equation

9 $$k=\frac{{\left(D_{50}^{2}\times n^{3}\right)}^{2.4}}{4000}$$

**Figure 10:**
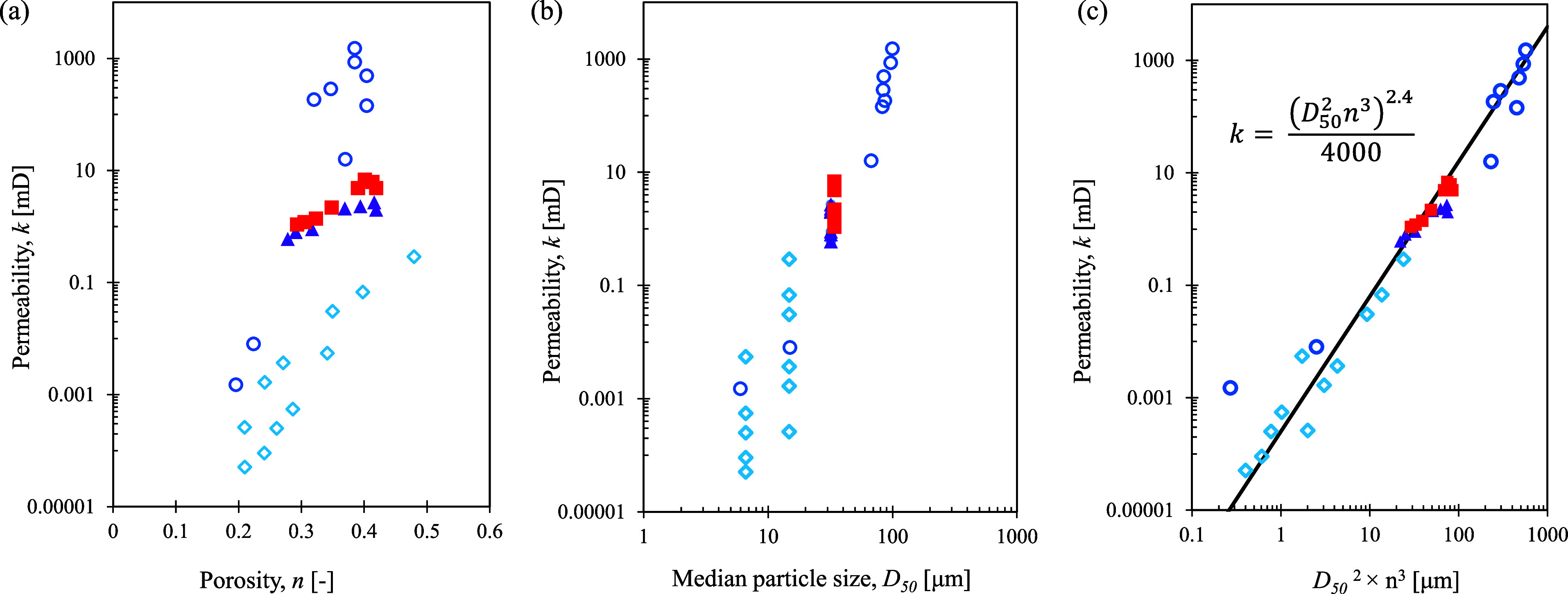
Vertical permeability in cores tested in this study (solid triangles for core 7P-2E and solid squares for core 20P-4E), in ref 49 (open diamonds for core 14P-2e), and in ref 62 (open circles for cores 16P-3, 17P, and 19P), all recovered from the Alaska site. (a) Permeability vs porosity. (2) Permeability vs *D*_50_. (c) Permeability vs (*D*_50_^2^ × *n* ^3^), where the solid line shows the prediction of the inset equation.

Although there are benefits in precise control of hydrate saturation, similar pore habits to that of methane hydrate in a water-saturated environment, and the simplification of the testing conditions when using THF hydrate as a proxy, no studies have directly compared the distribution and morphology of THF hydrate versus methane hydrate in the same sediment cores, nor their impact on sediment fabric due to hydrate formation and/or dissociation. The same core with THF hydrate showed comparable mechanical properties but 1 order of magnitude higher permeability compared to that with methane hydrate at identical effective porosity.^28^ Direct comparisons of fundamental chemical and physical properties between THF hydrate and methane hydrate are available,^34,39^ yet scarce between THF hydrate in sediment and methane hydrate in sediment. In addition, compared to the dissociation of methane hydrate, THF hydrate dissociation does not produce gas and thus is relatively less disruptive to the sediment fabric.

## 5 Conclusions

The depressurization process used for extracting methane from gas hydrate reservoirs causes significant changes in the mechanical and hydraulic properties of the sediments. For the extended-duration gas production test in northern Alaska, it was predicted in this laboratory study that a uniform in situ pore pressure decrease, for instance, from 9 to 2 MPa across the entire B1 sand reservoir, could cause a compaction of up to 1.0 m (5% of the initial thickness) in the overall reservoir thickness.

The laboratory-derived permeability anisotropy in the hydrate-free sediments at in situ stress shows a *k*_h_/*k*_v_ ratio of 3.0 to 5.0, slightly higher than the laboratory-predicted hydrate-bearing sediments’ permeability anisotropy of *k*_h_/kv 2.4 to 3.0 at the in situ stress (7 MPa for core 7P-2E and 9 MPa for core 20P-4). With increasing effective stress, the horizontal permeability decreases relatively more when compared to the vertical permeability because the cross-sectional area for horizontal fluid flow experiences a greater reduction during compaction than does the vertical flow area. The vertical permeability for multiple cores from the site can be well characterized by $k=\frac{{\left(D_{50}^{2}\times n^{3}\right)}^{2.4}}{4000}$ , where *D*_50_ and *n* are the median particle size and porosity, respectively.

The increase in permeability with hydrate dissociation and the decrease in permeability due to compaction from the increased effective vertical stress compete with each other during gas production from hydrates, as induced by depressurization. The zero-horizontal permeability-change curves generated for the D1 and B1 sands at the ANS long-term gas production site in this study show that the hydrate-free horizontal permeability of both units after depressurization should be greater than the horizontal permeability of the initial hydrate-filled reservoir, that is, the permeability increase due to hydrate loss surpasses the compaction-induced permeability decrease.

## ∥

790 Atlantic Dr NW, Atlanta, GA 30332, USA
